# Supplementary material for: Clustered rapid induction of apoptosis limits ZIKV and DENV-2 proliferation in the midguts of Aedes aegypti
Source: Commun Biol. 2021 Jan 15;4:69. doi: 10.1038/s42003-020-01614-9 (PMC7810730; doi:10.1038/s42003-020-01614-9)
Supplement: Supplementary file 1 — Supplementary Information [file 42003_2020_1614_MOESM1_ESM.pdf]

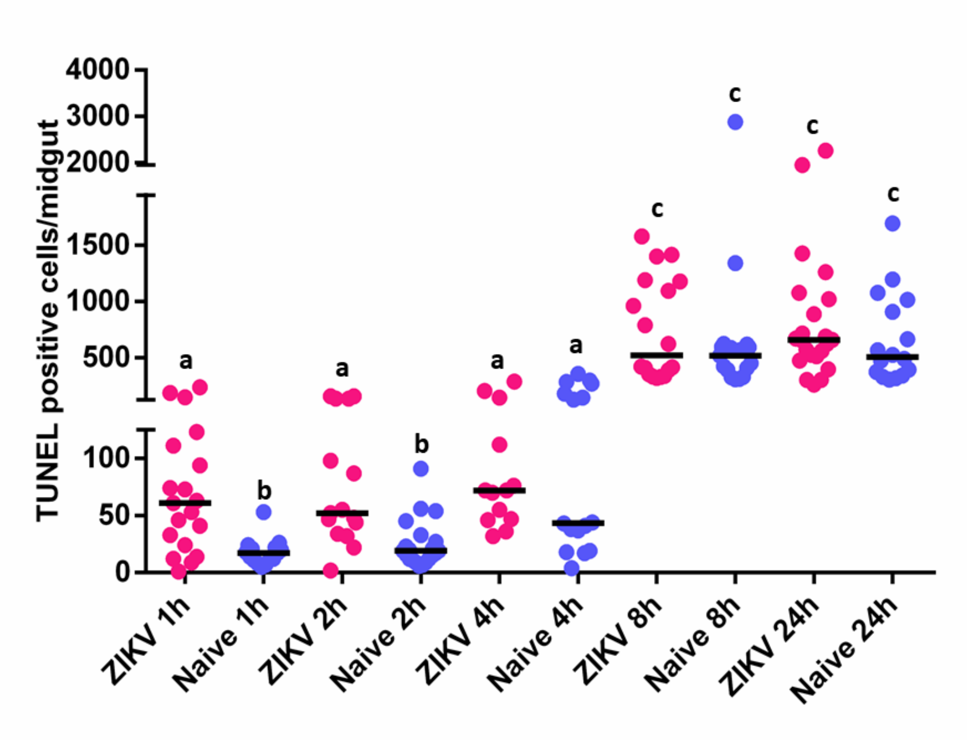

Supplementary Figure 1: Adult female ORL strain mosquitoes were fed a naive or ZIKV infected bloodmeal. Midguts were dissected and processed for TUNEL at the indicated times post infection. By 8 hours post-feeding, both naive and virus-fed groups have equivalently high levels of apoptotic cells, likely representing normal turnover of the gut epithelium in the process of blood digestion. An increase in TUNEL positive cells in virus fed mosquitoes is seen between 1 and 4 hours post-feeding (*n* (ZIKV 1h) = 19; *n* (naive 1h) = 15; *n* (ZIKV 2h) = 15; *n* (naive 2h) = 21; *n* (ZIKV 4h) = 13; *n* (naive 4h) = 16; *n* (ZIKV 8h) = 18; *n* (naive 8h) = 17; *n* (ZIKV 24h) = 22; *n* (naive 24h) = 15). Data is pooled from 3 independent replicates. Treatments without a common letter were found to be statistically significant ($\alpha = 0.05$) as calculated by Kruskal-Wallis test with Mann-Whitney post-hoc test.

	N	% Mortality	*p-value* [a]
**ZIKV**	78	8.5	–
**ZIKV+ hAAT**[b]	77	12.9	p = 0.1827
**DENV-2**	59	15.4	–
**DENV-2 + hAAT**[b]	77	6.5	p = 0.6368

[b]Concentration=10 mg/mL

[a]Calculated by χ2

Supplementary Table 1: Adult female ORL mosquitoes were fed a ZIKV or DENV-2 infected blood meal with or without 10 mg/mL hAAT and mortality was assessed at 7 days post infection during harvest for plaque assay. hAAT treatment did not cause any observable increase in mortality. Data is pooled from three replicates and significance was assessed at α=0.05 via a chi-squared test.

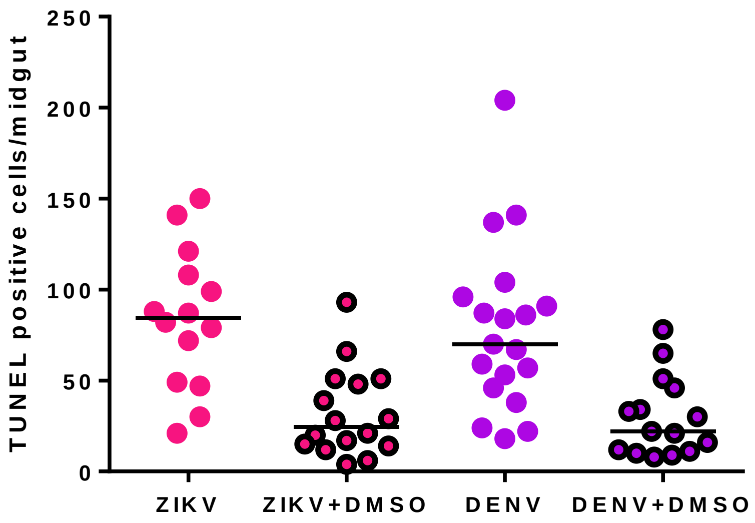

Supplementary Figure 2: Midguts treated with DMSO vehicle during *ex vivo* infection show reduced rapid induction of apoptosis. Midguts were dissected from adult female non-bloodfed ORL mosquitoes and infected *ex vivo* with plain virus stock or virus stock plus 1% molecular biology grade DMSO (Millipore-Sigma D2438-10mL). Data is pooled from 2 independent replicates. Horizontal line represents median. (*n* (ZIKV) = 14, *n* (ZIKV + DMSO) = 16, *n* (DENV) = 19, *n* (DENV + DMSO) = 15).

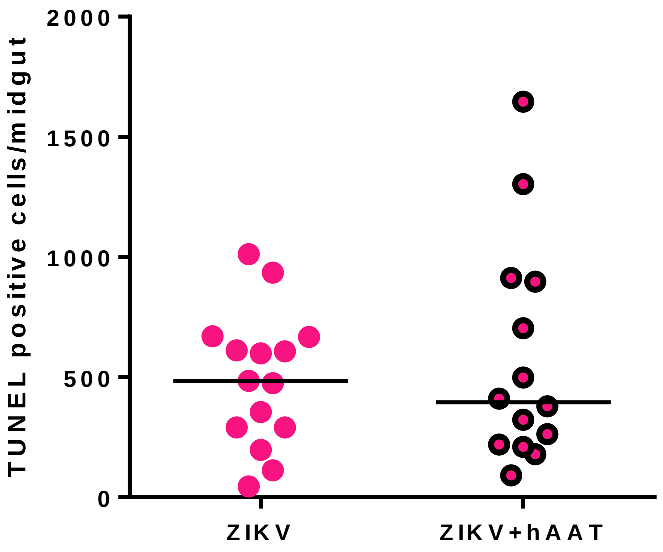

Supplementary Figure 3: Apoptosis inhibition from hAAT subsides by 24h post-infection. Adult female ORL mosquitoes were fed a blood meal containing 10^6 PFU/mL with or without 10mg/mL hAAT supplement. Midguts were dissected and prepared for TUNEL at 24h post-infection. Data is pooled from 2 independent replicates. (*n* (naïve) = 15, *n* (ZIKV + hAAT) = 14). Horizontal line indicates median.

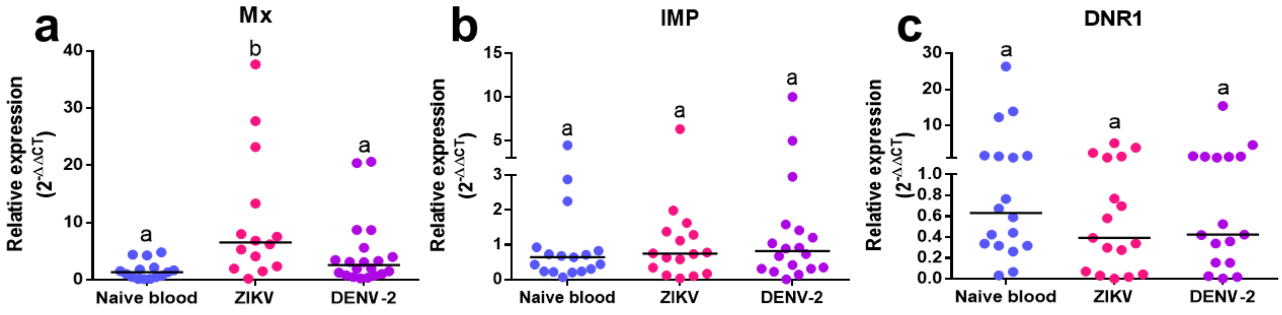

Supplementary Figure 4: Transcript level of IAP-antagonist *mx* is significantly increased during the RIA response to ZIKV infection, *IMP* and negative apoptosis regulator *DNR1* are not. Pools of 3 midguts were dissected from naive blood, ZIKV, or DENV-2 fed ORL strain female mosquitoes at 2hpi. Transcript expression level of *Mx* **(a)**, *IMP* **(b)** or *DNR1* **(c)**, was analyzed by RT-qPCR relative to *RPL32*. Data is pooled from 3 independent replicates (4 replicates in the case of *mx*). Treatments without a common letter were found to be statistically significant ($\alpha = 0.05$) as calculated by Kruskal-Wallis test with Mann-Whitney post-hoc test. Black line indicates median. (*n* (*mx* naive blood) = 15, *n* (*mx* ZIKV) = 14, *n* (*mx* DENV-2) = 20, *n* (*IMP* naive blood) = 17, *n* (*IMP* ZIKV) = 16, *n* (*IMP* DENV-2) = 18, *n* (*DNR1* naive blood) = 18, *n* (*DNR1* ZIKV) = 17, *n* (*DNR1* DENV-2) = 17).

Accession #	Gene Name	Primer Sequence
AAEL014196	*Michelob_x*	F: CGCCCCTCTAATATCCCTGT
		R: TTTCAAGAAGCGTCAAACCA
AAEL000590	*DNR1*	F: CATGAACGAAGAGCATGAGG
		R: GTAATGGCCCGGTAGAGACA
AAEL004392	*IMP*	F: GCAGAGTTATCAGTCGCAGC
		R: CCGCTTACACCACTTTCACC
NC_012532.1	*ZIKV polyprotein coding region*	F: GCCATCTATGCTGCCTTGAC
		R: ACTCCAAAGTCCCATGCGTA
NC_001474.2	*DENV-2 polyprotein coding region*	F: TGGTAGACAGAGGATGGGGA
		R: TTCCTTGCCGTGTTTTCCTG
AAEL003396	*60S Ribosomal protein L32*	F:CAGTCCGATCGCTATGACA
		R: ATCATCAGCACCTCCAGCTC

Supplementary Table 2: RT-qPCR primer sequences, gene names, and accession numbers.
