# Supplementary material for: Clustered rapid induction of apoptosis limits ZIKV and DENV-2 proliferation in the midguts of Aedes aegypti
Source: Commun Biol. 2021 Jan 15;4:69. doi: 10.1038/s42003-020-01614-9 (PMC7810730; doi:10.1038/s42003-020-01614-9)
Supplement: Supplementary file 2 — Description of Supplementary Files [file 42003_2020_1614_MOESM2_ESM.pdf]

Description of Additional Supplementary Files

File name: Supplementary Data 1

Description: This file is organized into separate tabs for each figure panel in the manuscript, titled Figure 1A through Supplementary Figure 4. Replicates are color coded with green cells for replicate 1, blue cells for replicate 2, and tan cells for replicate 3. TUNEL results for each figure are reported as TUNEL positive cells per midgut, counted by eye. Rt-qPCR results are reported as ΔCT calculated as viral genome cycle threshold (CT) minus 60S ribosomal protein L32 for Figure 4D and 4E, and reported as relative expression (2^-ΔΔCT, where ΔΔCT = (global average naive blood gene of interest CT - RPL32 CT) - (Sample gene of interest CT - RPL32 CT)) for Supplementary Figure 4. Plaque assay results were reported as plaque forming units per midgut.
