# Supplementary material for: Clustered rapid induction of apoptosis limits ZIKV and DENV-2 proliferation in the midguts of Aedes aegypti
Source: Commun Biol. 2021 Jan 15;4:69. doi: 10.1038/s42003-020-01614-9 (PMC7810730; doi:10.1038/s42003-020-01614-9)
Supplement: Supplementary file 4 — Reporting Summary [file 42003_2020_1614_MOESM4_ESM.pdf]

nature research

Corresponding author(s): Lei Zhou

Last updated by author(s): Nov 8, 2020

Reporting Summary

Nature Research wishes to improve the reproducibility of the work that we publish. This form provides structure for consistency and transparency in reporting. For further information on Nature Research policies, see our Editorial Policies and the Editorial Policy Checklist.

Statistics

For all statistical analyses, confirm that the following items are present in the figure legend, table legend, main text, or Methods section.

n/a	Confirmed	
☐	☒	The exact sample size (n) for each experimental group/condition, given as a discrete number and unit of measurement
☐	☒	A statement on whether measurements were taken from distinct samples or whether the same sample was measured repeatedly
☐	☒	The statistical test(s) used AND whether they are one- or two-sided<br>*Only common tests should be described solely by name; describe more complex techniques in the Methods section.*
☒	☐	A description of all covariates tested
☐	☒	A description of any assumptions or corrections, such as tests of normality and adjustment for multiple comparisons
☐	☒	A full description of the statistical parameters including central tendency (e.g. means) or other basic estimates (e.g. regression coefficient) AND variation (e.g. standard deviation) or associated estimates of uncertainty (e.g. confidence intervals)
☐	☒	For null hypothesis testing, the test statistic (e.g. F, t, r) with confidence intervals, effect sizes, degrees of freedom and P value noted<br>*Give P values as exact values whenever suitable.*
☒	☐	For Bayesian analysis, information on the choice of priors and Markov chain Monte Carlo settings
☒	☐	For hierarchical and complex designs, identification of the appropriate level for tests and full reporting of outcomes
☒	☐	Estimates of effect sizes (e.g. Cohen's d, Pearson's r), indicating how they were calculated

Our web collection on statistics for biologists contains articles on many of the points above.

Software and code

Policy information about availability of computer code

Data collection	Images were captured using BZ-X Viewer v1.3.1.1 and Openlab v5.5.3. qPCR data acquisition was performed using Applied Biosystems 7500 Software v2.0.1.
Data analysis	Statistical analysis was done using RStudio Desktop Open Source Edition and R version 4.0.0. Image analysis was performed using BZ-X Analyzer v1.3.1.1.

For manuscripts utilizing custom algorithms or software that are central to the research but not yet described in published literature, software must be made available to editors and reviewers. We strongly encourage code deposition in a community repository (e.g. GitHub). See the Nature Research guidelines for submitting code & software for further information.

Data

Policy information about availability of data

All manuscripts must include a data availability statement. This statement should provide the following information, where applicable:

- Accession codes, unique identifiers, or web links for publicly available datasets
- A list of figures that have associated raw data
- A description of any restrictions on data availability

Figures 1-4 and Extended Data Figures 1-4 have associated raw data. All relevant data is included in the manuscript as a supplementary source data file.

Field-specific reporting

Please select the one below that is the best fit for your research. If you are not sure, read the appropriate sections before making your selection.

[x] Life sciences [ ] Behavioural & social sciences [ ] Ecological, evolutionary & environmental sciences

For a reference copy of the document with all sections, see nature.com/documents/nr-reporting-summary-flat.pdf

Life sciences study design

All studies must disclose on these points even when the disclosure is negative.

Sample size	Sample sizes were determined by amount of tissue that can be processed in a single technical replicate of the assay, or by feeding rates of mosquitoes in the experimental groups. Sample sizes were deemed sufficient as comparable sample sizes were used in previously published studies on the discipline and are considered standard methodology in the discipline.
Data exclusions	No data was excluded.
Replication	Experiments shown in extended data figures 2 and 3 were replicated twice. All other experiments were replicated 3 times. There were no unsuccessful attempts at replication.
Randomization	Allocation of organisms into experimental groups was pseudorandom- female mosquitoes were sorted from males in no particular order and allocated into cups, which were then assigned to treatments in also in no particular order.
Blinding	Blinding was not performed due to personnel limitations.

Reporting for specific materials, systems and methods

We require information from authors about some types of materials, experimental systems and methods used in many studies. Here, indicate whether each material, system or method listed is relevant to your study. If you are not sure if a list item applies to your research, read the appropriate section before selecting a response.

Materials & experimental systems

n/a	Involved in the study	
[x]	[ ]	Antibodies
[x]	[ ]	Eukaryotic cell lines
[x]	[ ]	Palaeontology and archaeology
[ ]	[x]	Animals and other organisms
[x]	[ ]	Human research participants
[x]	[ ]	Clinical data
[x]	[ ]	Dual use research of concern

Methods

n/a	Involved in the study	
[x]	[ ]	ChIP-seq
[x]	[ ]	Flow cytometry
[x]	[ ]	MRI-based neuroimaging

Animals and other organisms

Policy information about studies involving animals; ARRIVE guidelines recommended for reporting animal research

Laboratory animals	Female adult (5-14 days post-eclosion) Orlando (ORL) strain Aedes aegypti mosquitoes
Wild animals	N/A
Field-collected samples	N/A
Ethics oversight	No ethical approval is required for invertebrates

Note that full information on the approval of the study protocol must also be provided in the manuscript.
